# Protein-based oleogels: effect of ultrasound on the structural characteristics of egg white-rapeseed dual-protein system^☆^

**DOI:** 10.1016/j.ultsonch.2026.107992

**Published:** 2026-08-06

**Authors:** Zhenlian Liao, Haojun Han, Junkun Ma, Yisha Xie, Xi Cao

**Affiliations:** a Chongqing Key Laboratory of Specialty Food Co-Built by Sichuan and Chongqing, School of Food and Bioengineering, Xihua University, Chengdu 610039, Sichuan, China; b College of Pharmacy and Food, Southwest Minzu University, Chengdu 610225, Sichuan, China

**Keywords:** Oleogel, Ultrasound modification, Rapeseed protein, Egg white protein, Microgel

## Abstract

As emerging fat substitutes, oleogels are widely recognized for their potential applications in food nutrition and health. In this study, the protein-based oleogel was prepared by oil absorption using egg white (EW) protein microgel and egg white-rapeseed (ER) dual-protein microgel. The microgel was obtained through ultrasound treatment (0–600 W) of the protein system, heating, freeze-thawing, ethanol solvent replacement, atmospheric drying, and ball milling pulverization. This study aimed to explore the effects of protein components and ultrasound powers on hydrogels obtained by heating the protein system, microgels, and oleogels. The results demonstrated that the addition of rapeseed protein increased the surface hydrophobicity of the hydrogel, leading to a lower contact angle of ER_0_ microgel (40.45°) than EW_0_ microgel (46.54°), and the oil absorption capacity (OAC) of ER_0_ microgel was higher than that of EW_0_ microgel by 20.75%. The ER_0_ oleogel exhibited a higher storage modulus (G’) than the EW_0_ oleogel. Additionally, the ultrasonic cavitation effect induced protein unfolding and hydrophobic group exposure, enhancing the surface hydrophobicity of ER hydrogel and reaching a maximum at 500 W. The enhanced hydrophobicity improved the lipophilicity of the microgels. Accordingly, the contact angle of ER_500_ microgel decreased to 31.97°, accompanied by the maximum OAC (196.00%). The ER_500_ oleogels displayed predominantly solid-like behavior (G' > G'') with a uniform, dense network microstructure. However, excessive ultrasound power induced protein aggregation, reducing the OAC of ER_600_ to 144.10%. This study provides insights for designing protein-based oleogels using an ultrasound-modified protein system, offering a promising strategy for food applications.

## Graphical abstract

**Figure:**
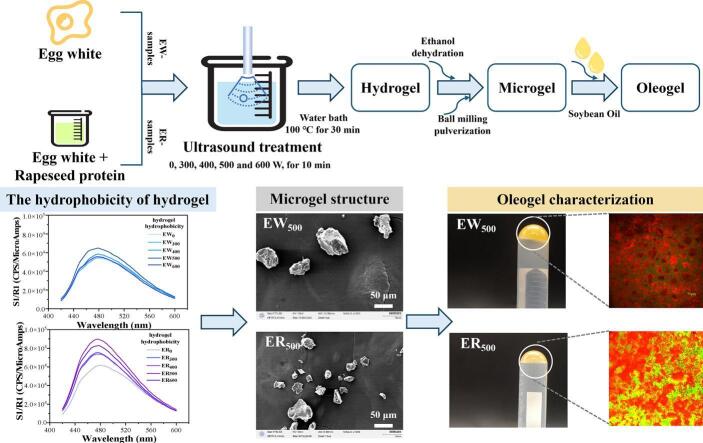

## 1 Introduction

Fat is an important source of nutrition and energy for humans and also significantly impacts the texture, taste, and flavor of foods [1]. Animal fats contain a high proportion of saturated fatty acids, which can be harmful to human health when consumed in excess. In contrast, vegetable oils have a more desirable fatty acid composition with higher unsaturated fatty acid content, making them frequent substitutes for animal fats [2]. However, vegetable oils are typically liquid at room temperature, and using them directly as replacements for solid fats may compromise food texture [3]. To address this issue, solidification processes are employed to convert liquid vegetable oils into solid forms. Oleogelation is an innovative approach to oil solidification, in which three-dimensional network structures are constructed *via* molecular self-assembly or crystallization-induced solidification [4], physically entrapping oils within a semi-solid system.

Compared with conventional solid fats, oleogelation structures oil *via* physical entrapment rather than chemical modification, avoiding the formation of trans fatty acids and preserving unsaturated fatty acids [5]. Oleogel preparation methods include direct and indirect methods. Direct methods rely on beeswax [6], ethyl cellulose[7], and sterol compounds [8] as oleogelators in the oil phase to build a threedimensional network [9]. However, direct methods often require high temperatures, which may cause oil oxidation and nutrient degradation[10], and these materials are often inedible or nutritionally deficient [11]. Indirect methods include emulsion templating, foam templating, and aerogel templating [12], which usually do not involve heating. Among them, the aerogel templating approach is promising due to its operational simplicity, requiring only the immersion of aerogels in oil to obtain oleogels[13]. Recently, direct oil absorption has emerged as a modified strategy of aerogel templating. Compared with aerogel templating *via* direct immersion of bulk aerogels into oil, direct oil absorption employs ball milling to comminute porous gels into microgel particles as oleogelators before dispersion in oil, offering superior mechanical stability, logistical convenience, and operational simplicity [14].

Proteins, as natural macromolecules, are characterized by wide availability, high nutritional value, and amphiphilicity [13]. Li et al. [15] reported that oleogels prepared with whey protein isolate as the oleogelator exhibited favorable textural and stability properties. Recent studies have demonstrated that protein blending can further enhance gel properties. Li et al. [16] found that bamboo shoot protein (BSP) and soy protein isolate (SPI) formed a gel network via hydrogen bonding, and the resulting BSP/SPI (4:1) oleogel exhibited superior viscosity recovery and cohesiveness compared to the pure soy protein isolate counterpart. Similarly, Huang et al. [17] found that the appropriate addition of egg white protein facilitated the unfolding of eel myofibrillar protein molecules, exposing additional hydrophobic groups and sulfhydryl sites. This effect strengthened hydrophobic interactions and disulfide cross-linking, ultimately yielding a composite gel with greater strength than that of pure myofibrillar protein. These findings indicate that protein blending can modulate gel network structures by promoting protein unfolding and enhancing intermolecular interactions, consequently improving oleogel performance. Egg white protein has a high nutritional value and multiple functional properties, including gelation and foaming properties, rendering it an ideal material for the preparation of oleogels [18]. Liu et al. [14] successfully prepared oleogels using egg white protein as the sole oleogelator. Rapeseed protein, obtained as a by-product of rapeseed oil extraction, is abundant in hydrophobic amino acids, conferring superior lipophilicity [19]. Blending the two proteins is anticipated to further enhance the oil retention stability and gel properties of oleogels prepared solely with egg white.

The hydrophobic moieties within protein molecular architectures can be exposed by physical modification, enhancing surface hydrophobicity and lipophilicity, improving oil absorption capacity as oleogelators. Ultrasound treatment serves as a green physical modification technique [20], disrupting the compact protein structure *via* cavitation, exposing hydrophobic groups [21]. Saneei et al. [22] confirmed that ultrasound treatment enhanced the surface hydrophobicity and foam stability of soy protein, with the ultrasound-treated group exhibiting significantly higher oil holding capacity in oleogels than the untreated group. Yu et al. [23] showed that ultrasound treatment elevated the oil holding capacity of oleogels prepared with soy protein isolate and phosphatidylserine to 95.3%, significantly enhancing thermal stability. Li et al. [24] prepared oleogels from candelilla wax and nut oil, and found that high-intensity ultrasound (95 W, 10 s) markedly improved rheological properties (higher G' and G''), oil binding capacity, and hardness. Thus, ultrasound treatment augments protein surface hydrophobicity, enhances interfacial stability, and facilitates gel network assembly, rendering it a promising approach for sophisticated protein-based oleogel preparation.

Currently, it has not been reported that a comparison of oleogel quality between single-protein and dual-protein systems under ultrasound treatment, and the influence of ultrasound power on the properties of dual-protein oleogel has not been examined. In this study, protein-based oleogels were prepared using egg white (EW) protein and egg white-rapeseed (ER) dual protein. The rheological characteristics and microstructure of EW and ER oleogels treated at 0–600 W ultrasound power were determined. The effects of protein hydrophobicity, hydrogel network structure, and microgel interfacial properties on oleogel characteristics were explored. This research proposes a novel protein-based approach for oleogel preparation, offering new insights for the development of low-saturated-fat solid fat alternatives.

## 2 Materials and methods

### 2.1 Materials

Soybean oil was obtained from Yi Hai Guangzhou Cereal and Oil Industry Co., Ltd. (Guangzhou, China). The rapeseed protein isolate (food-grade, 8.42% moisture content) was sourced from Shaanxi West Peptide Biotechnology Co. (Xianyang, China). Fresh eggs were sourced from a local supermarket (Wal-Mart, Chengdu, China). Nile Red (HPLC, ≥95%) and ANS fluorescent probe (>97%) were obtained from Aladdin Reagent Company (Shanghai, China). Ethanol (AR, ≥99.7%) and Nile Blue A (AR, 75%) were obtained from Chuandong Chemical Co., Ltd. (Chongqing, China). Hydrochloric acid (AR) was obtained from Jinshan Chemical Reagent Co., Ltd. (Chengdu, China). Sodium hydroxide (AR) was purchased from Hualikexi Instrument Co., Ltd. (Chengdu, China). All the reagents utilized in the research were of analytical grade.

### 2.2 Purification of rapeseed proteins

Defatted rapeseed powder (30.0 g) was dispersed in deionized water (600 mL). The pH was elevated to 11.0 using 2 mol/L NaOH, and the mixture was stirred for 2 h. Subsequently, the solution was centrifuged (4 °C, 8000 r/min, 20 min) in a benchtop high-speed centrifuge (H1750R, Xi'an Yidian Instruments Co., Ltd., China). The pH of the collected supernatant was readjusted to 4.5 with HCl (1 mol/L). After a second centrifugation, the precipitate was collected as the purified rapeseed protein, and the pH was neutralized to 7.0 using NaOH (2 mol/L). Then, the purified protein was freeze-dried at −40 °C for 48 h using a freeze dryer (FDU-1100, Shanghai Ailang Instrument Co., Ltd., China), yielding purified rapeseed protein, which was then stored at 4 °C for subsequent analysis. The protein purity was 75.695%, determined by the Kjeldahl method with 5.53 of K value.

### 2.3 Protein modified by ultrasound

The egg white (45 mL) was diluted with deionized water (15 mL), and the EW protein solution was obtained with a total solids content of 11.21% (w/v). The egg white (45 mL) was mixed with 15 mL of rapeseed protein solution (3%, w/v), and the ER dual protein solution was obtained with a total solids content of 11.86% (w/v). The ER solution contained egg white and rapeseed protein at a mass ratio of approximately 15:1. To fully mix the protein solution, the samples were stirred with a high-speed disperser (T25DS25, IKA, Germany) at 5000 r/min for 5 min. Then, the samples were subjected to ultrasound treatment with an ultrasonic cell disruptor (S-JY92-IIN, Shanghai Sheyan Instruments Co., Ltd., China) equipped with a 6-mm-diameter titanium microtip. The ultrasonic frequency was 20–25 kHz with automatic frequency tracking. Ultrasound was performed in pulse mode (on-time: 1.0 s; off-time: 1.0 s; duty cycle: 50.0%) at nominal instrument power of 0, 300, 400, 500, and 600 W for 10 min. Based on the sample volume of 60 mL, the corresponding energy densities were 0, 5.0, 6.7, 8.3, and 10.0 W/mL. The corresponding actual energy delivered was 0, 90.0, 120.0, 150.0, and 180.0 kJ, respectively. The probe tip was immersed 1 cm below the liquid surface without contacting the bottom of the vessel. An ice-water bath was used to keep the temperature at 30 °C during ultrasound treatment.

### 2.4 Preparation of hydrogels, microgels, and oleogels

**Fig. 1:**
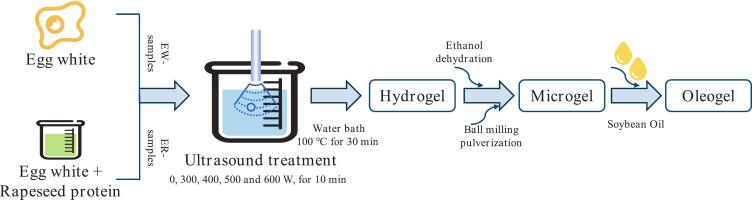
The preparation processes of hydrogel, microgel, and oleogel.

The preparation processes of hydrogels, microgels, and oleogels are illustrated in Fig. 1.

The hydrogel was prepared by heating the ultrasound-modified protein solution for 30 min at 100 °C [25]. The hydrogels were diced into small blocks. All samples were then frozen at −20 °C for 24 h, followed by thawing at room temperature. A gradient ethanol exchange method was employed to remove water from the hydrogels. Samples were sequentially immersed in ethanol (0.5, 1.5, and 2.5 h) at a hydrogel-to-ethanol ratio of 1:10 (w/v). After drying under a fume hood for 12 h, the sample was ovendried at 55 °C until the weight remained unchanged. The dried ethanol gel was obtained.

The preparation of microgel was based on the method reported by Liu et al. [14] with modifications. The ethanol gel was milled with 10-mm-diameter beads at a bead-to-sample ratio of 8:1 in a ball mill (MM440, Laixis Co., Ltd., China) operating at 20 r/min for 1.5 min to obtain EW microgels and ER microgels.

The oleogel was prepared using the oil absorption method. Briefly, microgels (2 g) were dispersed in soybean oil (20 g) and homogenized at 10,000 rpm for 3 min. Unabsorbed oil was removed by centrifugation at 8,000 r/min for 15 min at 4 °C, and the procedure was repeated twice to yield oleogels.

### 2.5 Ultrasound-modified protein characterization

#### 2.5.1 Surface hydrophobicity of proteins

Surface hydrophobicity of the proteins was measured by the F-4600 fluorescence spectrophotometer. The protein solution was diluted to 0.2 mg/mL, followed by the addition of 20 µL of 8-anilino-1-naphthalenesulfonic acid (ANS) solution (8 mM). After incubation for 10 min in dark conditions, fluorescence intensity was recorded with excitation at 390 nm and emission scanned from 470 to 600 nm [25].

#### 2.5.2 Intrinsic fluorescence of proteins

The intrinsic fluorescence of proteins was measured by a F-4600 fluorescence spectrophotometer (Hitachi, Japan). Following dilution of the protein stock solution to 1 mg/mL, fluorescence intensity was acquired with excitation at 280 nm and emission collected over 290–450 nm [26]. The slit width was set to 3 nm for both excitation and emission.

### 2.6 Hydrogels characterization

#### 2.6.1 Surface hydrophobicity of hydrogels

The surface hydrophobicity of the hydrogels was measured using an ANS fluorescent probe on a spectrofluorometer (F-4600, Hitachi, Japan), adapted from the method of Yan et al. [27]. Following the preparation described in Section 2.4, half of the hydrogel sample was immersed in ultrapure water (20 mL). The mixture was then homogenized at 5000 rpm for 3 min and subsequently centrifuged at 8000 rpm for 10 min, and the supernatant was collected. The supernatant (0.25 mL) was diluted with biuret reagent (1:10, *v/v*), and protein content was determined using a SpectraMax i3x microplate reader (Molecular Devices, USA). All sample solutions were then diluted to a final concentration of 0.2 mg/mL. After the addition of 20 µL of ANS solution to 3 mL of the diluted sample solution (3 mL), the resulting solution was vortexed and incubated for 10 min in dark conditions. To measure the fluorescence intensity, the excitation wavelength was set to 390 nm, and emission spectra were collected over the 470–600 nm range with a slit width of 3 nm for both excitation and emission.

#### 2.6.2 Secondary structure of hydrogels

The secondary structure was determined using a circular dichroism spectrometer (Applied Photophysics, UK) as described by Zhou et al. [28] with minor modifications. The prepared hydrogel was added to deionized water (1:10, w/v), homogenized, and centrifuged. The supernatant was collected and diluted to 0.2 mg/mL. Subsequently, circular dichroism spectra of the sample were recorded over the wavelength range of 190–260 nm, with each measurement repeated three times. The protein secondary structure contents were deconvoluted using the Circular Dichroism spectra deconvolution program.

### 2.7 Microgels characterization

#### 2.7.1 Scanning electron microscopy (SEM) observation of microgels

The surface morphology of the microgel was examined by SEM (CIQTEK, SEM-3200A, China). The sample was evenly distributed onto a metal sample stage covered with conductive double-sided tape. After gold sputtering, the sample was imaged under SEM at 10 kV and × 1000 magnification [29]. Particle size analysis was performed on the resulting microgel images using ImageJ software.

#### 2.7.2 Three-phase contact angle of microgels

Three-phase contact angle (air-oil-particle) was measured using an SL200KS optical tensiometer (KINO, USA). A 1 mm sheet was prepared by pressing 100 mg of microgel, and 2 µL of soybean oil was then injected onto the surface with a syringe. Subsequently, the droplet images were captured with a camera. The contact angle was determined by image analysis using built-in software. All measurements were conducted at 25 °C, and each sample was measured at least twice [30].

#### 2.7.3 Oil absorption capacity of microgels

A small amount (about 1 g) of the microgel was weighed and then completely immersed in an excess of soybean oil (1:10, w/w). Subsequently, the mixture was stirred using the high-speed disperser (10000 rpm, 3 min), followed by centrifugation at 8000 rpm for 15 min at 4 °C. After the unabsorbed oil was discarded, this entire procedure was repeated twice, and the final oleogel was weighed. The following equation was used to calculate the oil absorption capacity (OAC) of the oleogel [13].

$$\text{OAC (\%)=}\frac{{\text{M}}_{\text{1}}\text{-}{\text{M}}_{\text{0}}}{{\text{M}}_{\text{0}}}\text{×100\%}$$

where M_0_ and M_1_ represent the masses of the microgel and oleogel, respectively.

### 2.8 Oleogels characterization

#### 2.8.1 Macroscopic appearance of oleogels

Macroscopic images of the oleogels were obtained by inverting the samples. Specifically, oleogels stored in 50 mL centrifuge tubes were placed upside down in a photo booth for photography.

#### 2.8.2 Microstructure of oleogels

The microstructure of the oleogels was observed via a confocal laser scanning microscope (CLSM600, Ningbo Sunny Instruments Co., Ltd, Ningbo, China), according to a modified procedure based on Yu et al. [31]. Equal amounts of Nile red and Nile blue were mixed to prepare a 1 mg/mL dye solution. After staining with 0.5 mL of the dye solution for 30 min, the oleogel sample was transferred onto a confocal dish and imaged under the CLSM. The excitation wavelengths for Nile Red and Nile Blue were 488 and 633 nm, respectively.

#### 2.8.3 Rheological properties of oleogels

The rheological properties of the oleogels were characterized using a MCR302 rotational rheometer (Anton Paar, Austria) fitted with a PP25 parallel plate geometry (25 mm diameter, 1 mm gap) at 4 °C. Before testing, a strain sweep (0.01–100% at 1 rad/s) was conducted to verify the linear viscoelastic region (LVR). A strain level of 1% was employed for the following frequency sweep. The viscoelastic properties of the oleogels were obtained via oscillatory frequency sweep tests (0.1–100 rad/s, 1% strain, 4 °C) [32], recording the storage modulus (G'), loss modulus (G"), and loss tangent (tan δ). Steady-state rheological measurements were performed to record changes in viscosity and shear stress of the oleogels over a shear rate range of 0.01 to 100 s^−1^.

### 2.9 Data analysis

Unless otherwise indicated, all experimental results are presented as mean ± standard deviation (SD) of three independent replicates. Statistical analysis was performed using SPSS 27 (IBM SPSS Statistics), with mean comparisons conducted *via* the Least Significant Difference (LSD) and Duncan tests. A 95% confidence level (p < 0.05) was adopted.

## 3 Results and discussion

### 3.1 Ultrasound-modified protein characterization

#### 3.1.1 Surface hydrophobicity and intrinsic fluorescence of protein

**Fig. 2:**
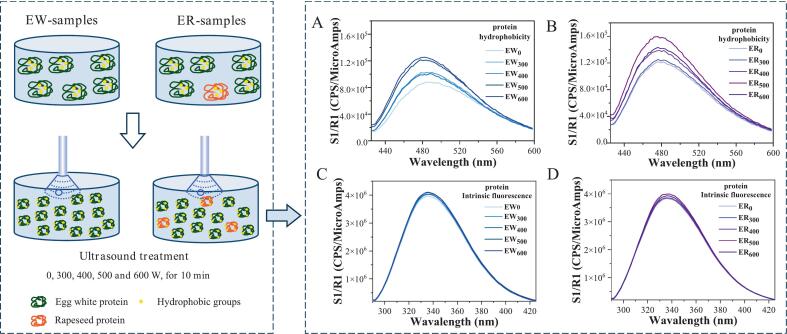
Surface hydrophobicity (A, B) and intrinsic fluorescence (C, D) of EW protein (A, C) and ER protein (B, D). *Note:* EW is egg white protein. ER is egg white-rapeseed protein.

The surface hydrophobic characteristics of proteins are primarily reflected in the extent of exposure of nonpolar amino acid residues to the aqueous environment [33], which essentially refers to the content of hydrophobic amino acid residues in the protein. An increase in the surface hydrophobicity of the protein indicates that the hydrophobic regions of the peptide and nonpolar side chains are exposed [34]. As shown in Fig. 2A and B, the surface hydrophobicity of the ER protein system was generally higher than that of the EW protein system, indicating that the addition of rapeseed protein provided more hydrophobic groups. Additionally, correlation analysis (Table S1 and S2) revealed that the surface hydrophobicity of ER and EW protein systems showed a positive correlation with the G' of the respective oleogels (r = 0.716 and 0.894*, respectively). As shown in Table S3, the maximum emission wavelength (λ_max_) decreased with increasing ultrasound power, while no statistically significant variation was detected across the power levels (p > 0.05). Conversely, the maximum ANS fluorescence intensity (F_max_) showed a significant increase with rising ultrasound power (p < 0.05). These results indicated that ultrasound treatment did not significantly alter the polar environment of the ANS-binding sites but significantly increased the number of accessible hydrophobic sites on the protein surface. This likely resulted from the shock waves and shear stresses released upon cavitation bubble collapse during ultrasound irradiation [35], which induced protein conformational changes and denaturation. Such denaturation induced protein unfolding and exposed hydrophobic moieties [36], promoting ANS binding to hydrophobic sites. In the ER protein system, the surface hydrophobicity of the samples peaked at 500 W and then declined as the power increased to 600 W, likely due to protein aggregation that buried hydrophobic groups within the protein interior. Consistently, Sun et al. [37] reported that increasing ultrasound power induced partial protein denaturation and subsequent reaggregation, decreasing surface hydrophobicity. At a deeper mechanistic level, the aggregates likely formed *via* intermolecular disulfide (S-S) cross-linking when the dual proteins were subjected to high-power ultrasound treatment [38]. These aggregates effectively shielded the exposed hydrophobic sites, reducing accessibility to the fluorescent probe.

Changes in protein tertiary structure can be detected by intrinsic fluorescence, which relies on the label-free intrinsic emission of aromatic residues (tryptophan, tyrosine, and phenylalanine). Tryptophan dominates the intrinsic fluorescence properties of proteins due to its higher extinction coefficient and quantum yield [39]. As shown in Fig. 2C and D, the overall intrinsic fluorescence intensity of the ER protein system was lower than that of the EW protein system, suggesting that interactions occurred between rapeseed protein and egg white protein, leading to changes in the microenvironment of some tryptophan residues or fluorescence quenching [40]. This result agrees with the findings of Xie et al. [41], who showed that the intrinsic fluorescence intensity of the rice-cod protein mixture was lower than that of the individual rice and cod proteins. They speculated that interactions between two different proteins led to the burial of hydrophobic groups within the protein interior, resulting in decreased intrinsic fluorescence intensity. Following ultrasound treatment, the intrinsic fluorescence intensity of both EW and ER protein systems increased significantly. This enhancement likely results from the intense shear forces generated by cavitation, which disrupt intramolecular hydrophobic interactions. Consequently, the tertiary structure unfolded, exposing tryptophan residues originally buried within the hydrophobic core to the polar environment [42]. The exposure of internal hydrophobic groups of the protein increased the surface hydrophobicity (as shown in Fig. 2A and B). However, excessive ultrasound power may induce protein over-aggregation, resulting in the reburial of exposed tryptophan residues within the aggregates. Consistently, the intrinsic fluorescence intensity of the ER protein system decreased when the ultrasound power reached 600 W.

### 3.2 Hydrogels characterization

#### 3.2.1 Surface hydrophobicity of hydrogels

**Fig. 3:**
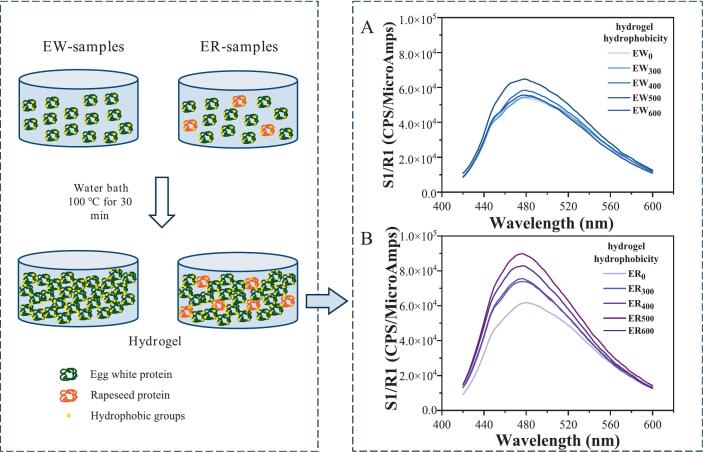
Surface hydrophobicity of EW hydrogel (A) and ER hydrogel (B). *Note:* EW is egg white protein. ER is egg white-rapeseed protein.

Surface hydrophobicity of the hydrogels was measured to assess the influence of protein composition and ultrasound treatment. Greater exposure of hydrophobic residues corresponds to higher surface hydrophobicity. Consistent with the findings on surface hydrophobicity in proteins (Table S3), the hydrogels exhibited a similar trend (Table S4). Although the λ_max_ decreased with increasing ultrasound power, the differences across power levels were not significant (p > 0.05), whereas the F_max_ increased significantly (p < 0.05). In addition, the surface hydrophobicity of ER hydrogels was significantly higher than that of EW hydrogels (Fig. 3), suggesting that rapeseed protein introduces additional hydrophobic groups. Within the range of 0–500 W, the surface hydrophobicity of both hydrogels increased with increasing ultrasound power. However, the increase in ANS fluorescence intensity was greater for the ER hydrogels (from 61686.46 to 88100.20) than for the EW hydrogels (from 54642.06 to 65589.16). This suggested that the intense shear force generated by ultrasonic cavitation induced protein unfolding, exposing more internal hydrophobic groups to the molecular surface in the dual-protein system. The enhanced surface hydrophobicity provided more hydrophobic binding sites for the subsequent formation of oleogels. Consistent with this result, Wang et al. [43] found that the surface hydrophobicity of bovine plasma proteincarboxymethyl cellulose composite gels was significantly enhanced by ultrasound treatment. The ANS fluorescence intensity of the hydrogels peaked at 500 W and declined at 600 W, consistent with the OAC results (Fig. 6C). The observed decrease was probably attributable to thermal effects that promoted protein aggregation, burying exposed hydrophobic groups within the aggregates [44]. Consequently, the surface hydrophobicity of the hydrogels decreased, reducing the OAC of the microgels.

#### 3.2.2 Secondary structure of hydrogels

**Fig. 4:**
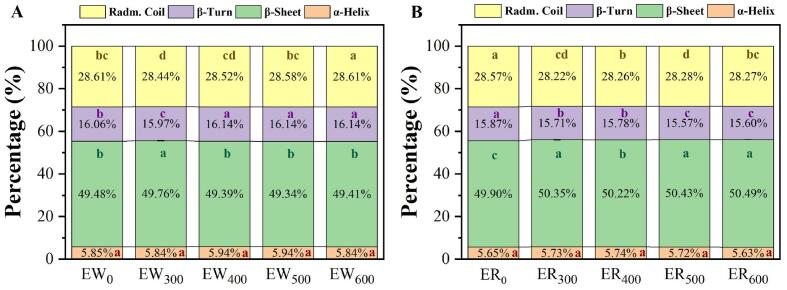
The secondary structure of EW hydrogel (A) and ER hydrogel (B). *Note:* EW is egg white protein. ER is egg white-rapeseed dual protein. Different letters indicate the significance (p < 0.05) among samples.

The secondary structure of the hydrogels is shown in Fig. 4. Overall, the β-sheet content in ER hydrogels was higher than that in EW hydrogels. The β-sheet is an ordered conformation stabilized by hydrogen bonds, and elevated β-sheet content generally reflects enhanced ordered intermolecular cross-linking among proteins[45]. In this study, the higher β-sheet content in ER hydrogels corresponded to their superior OAC (Fig. 6C). The addition of rapeseed protein strengthened intermolecular interactions within the dual-protein system and facilitated the formation of an ordered hydrogen-bonded network, enhancing the physical entrapment of oil within the gel matrix and improving the OAC of the microgels [46]. As the ultrasound power increased, the β-sheet content of the ER hydrogels increased significantly, accompanied by a marked decrease in β-turn and random coil contents. The shear forces generated by ultrasonic cavitation disrupted disordered regions of protein molecules and promoted the conversion of β-turns and random coils into β-sheets, resulting in a more stable cross-linked network structure. This trend aligns with findings on ultrasound-treated potato protein isolates, in which β-sheet content increased while random-coil and β-turn content decreased [47]. Liu et al. [38] found that ultrasound treatment increased the proportion of β-sheet structures in oat-whey dual-protein gels, indicating that ultrasound treatment formed a more ordered protein conformation within the gel. However, the β-sheet content of the EW hydrogels peaked at 300 W (49.76%) and then decreased. This might be because overheating weakened the ultrasound effect, leading to partial protein denaturation and a transition to random coil.

### 3.3 Microgels characterization

#### 3.3.1 Microstructure and particle size of microgels

**Fig. 5:**
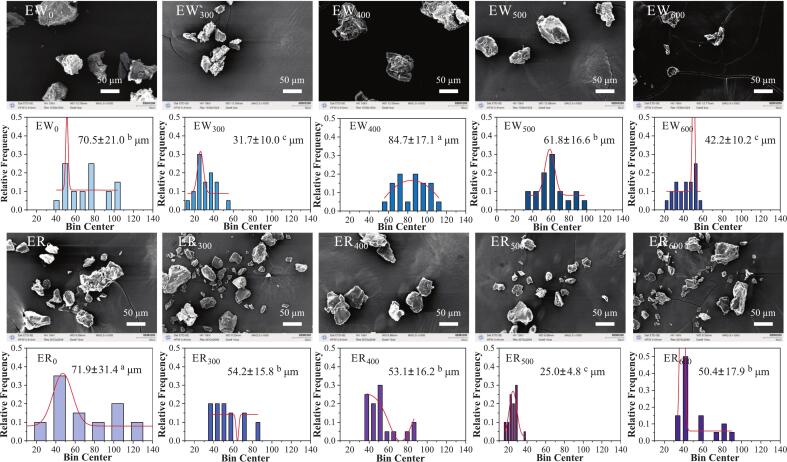
The microstructure and particle size distribution of EW microgel and ER microgel, with the mean particle size indicated in the upper right corner of the particle size distribution plots. *Note:* EW is egg white protein. ER is egg white-rapeseed dual protein. Different letters indicate the significance (p < 0.05) among samples.

**Fig. 6:**
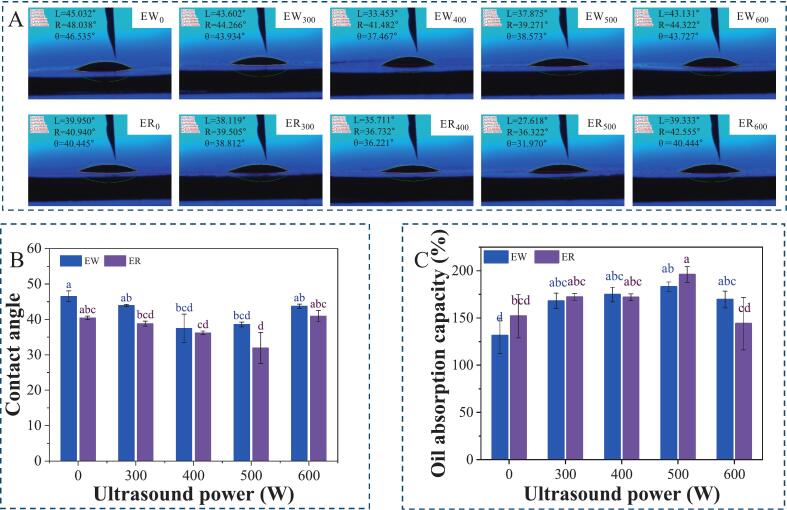
The three-phase contact angle (A, B) and oil absorption capacity (C)of EW microgel and ER microgel. *Note:* EW is egg white protein. ER is egg white-rapeseed dual protein. Different letters indicate the significance (p < 0.05) among samples.

The microstructure and particle size of the microgels are shown in Fig. 5. The EW microgels (EW_0_) and ER microgels (ER_0_) exhibited pronounced surface roughness characterized by extensive folds and porous structures. Following ultrasound treatment, the surface roughness of both microgels was markedly reduced, with an overall morphology trending toward smoothness. Cavitation-induced shear forces disrupted large protein aggregates, facilitating more uniform rearrangement and ordered assembly of protein molecules during subsequent heat-induced gelation [48]. The lipophilicity of microgels is associated with particle size. A smaller particle size corresponds to a larger specific surface area, facilitating greater exposure of hydrophobic groups on the surface. Without ultrasound treatment, ER microgels (ER_0_) displayed a broader particle size distribution and a larger mean particle diameter (130 μm) compared to EW microgels (110 μm for EW_0_). The increased particle size likely arose from the co-aggregation of rapeseed and egg white proteins during thermal gelation. Kuang et al. [49] observed large aggregates in pea protein-egg white protein mixed gel systems that were absent in pure pea protein systems, confirming the mutual aggregation of dual proteins. Without ultrasound treatment, although the addition of rapeseed protein increased the microgel particle size, the ER microgels exhibited superior OAC (Fig. 6C). Studies have shown that rapeseed protein is rich in hydrophobic amino acids and that heat treatment induces protein unfolding, exposing hydrophobic sites [19], which likely enhanced the OAC of the microgels. As ultrasound power increased, the particle size of both microgels first decreased and then increased, in agreement with the results reported by Wang et al. [50]. The strong shear force generated by ultrasonic cavitation disrupts large protein aggregates, reducing the particle size while simultaneously inhibiting protein aggregation [50]. However, excessively high ultrasound intensity caused excessive protein aggregation, resulting in larger particles with a broader size distribution [51]. Among all microgels, the ER_500_ exhibited the smallest and most uniform particle size, with a narrow distribution centered around 20 μm, which maximized its specific surface area and enhanced OAC. Additionally, correlation analysis (Table S1) showed that ER microgel particle size was negatively correlated with the G' of ER oleogels (r = -0.959**), indicating that smaller microgel particles contribute to a higher G' of oleogels.

#### 3.3.2 Three-phase contact angle and oil absorption capacity of microgels

The three-phase contact angle (air-oil-particle) reflects the lipophilicity of microgel components. A smaller contact angle indicates greater lipophilicity of the microgel surface, promoting oil spreading and adsorption, and consequently improving OAC [25]. As shown in Fig. 6A and B, the three-phase contact angle of ER microgels (ER_0_) decreased by 6.09° compared to EW microgels (EW_0_). This indicated that the dual-protein microgels exhibited greater lipophilicity and enhanced oil adsorption, consistent with the higher OAC of ER microgels relative to EW microgels (Fig. 6C). In addition, compared with the untreated samples, the contact angles showed a similar decreasing trend after ultrasound treatment. Studies have shown that ultrasonic cavitation can expose more hydrophobic groups on proteins [52], which enhanced the lipophilicity of the microgels. At 500 W, the contact angle of ER_500_ reached a minimum of 31.970°. However, further increasing ultrasound power disrupted the protein structure, as reflected by the contact angle of ER_600_ rising to 40.444°.

The OAC of microgels serves as one of the key determinants of oleogel quality. Fig. 6C illustrates the influence of ultrasound power and protein system on the OAC. Without ultrasound treatment, the OAC of the ER microgels (ER_0_, 152.05%) was significantly higher than that of the EW microgels (EW_0_, 131.3%), which indicated a stronger absorption capacity of the ER microgels. The addition of rapeseed protein facilitated intermolecular interactions within the dual-protein system, forming a more stable network structure with enhanced oil encapsulation capacity. Subsequently, as the ultrasound power increased, OAC of EW microgels increased from 131.3% (EW_0_) to 182.95% (EW_500_), and that of ER microgels increased from 152.05% (ER_0_) to 196% (ER_500_). This upward trend stemmed from ultrasound-induced partial unfolding of egg white and rapeseed proteins, exposing hydrophobic groups and enhancing hydrophobic interactions with the oil phase, improving OAC [53], [54]. However, when the ultrasound power was increased from 500 W to 600 W, the OAC of EW microgels decreased from 182.95% (EW_500_) to 169.6% (EW_600_), and the OAC of ER microgels decreased from 196% (ER_500_) to 144.1% (ER_600_). The increased three-phase contact angle (Fig. 6A and B) and enlarged particle size (Fig. 5) likely contributed to the OAC decrease.

### 3.4 Oleogels characterization

#### 3.4.1 Macroscopic appearance and microstructure of oleogels

**Fig. 7:**
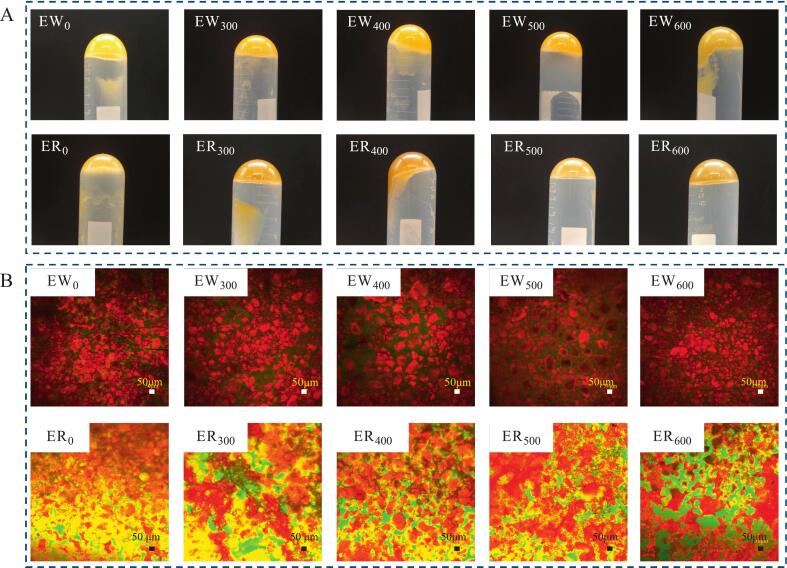
Macroscopic appearance (A) and microstructure (B) of EW oleogel and ER oleogel. *Note*: EW is egg white protein. ER is egg white-rapeseed dual protein.

Fig. 7A shows the color and state of the oleogels after inversion. All samples appeared as yellow, gel-like solids. EW oleogels exhibited a bright yellow color, while ER oleogels displayed a darker shade. It has been found that the rapeseed protein solution exhibited a green color [55], and adding rapeseed protein to the EW system could decrease the lightness of ER oleogels. In addition, as ultrasound power increased, the fluidity of EW oleogels first decreased and then increased. Among them, EW_500_ exhibited the lowest fluidity but still showed a tendency to flow. ER oleogels exhibited a similar trend but showed overall superior behavior compared to EW oleogels. Notably, ER_500_ remained firmly attached to the bottom of the inverted vial without flowing, indicating a stronger oil immobilization capacity of its gel network.

**Fig. 8:**
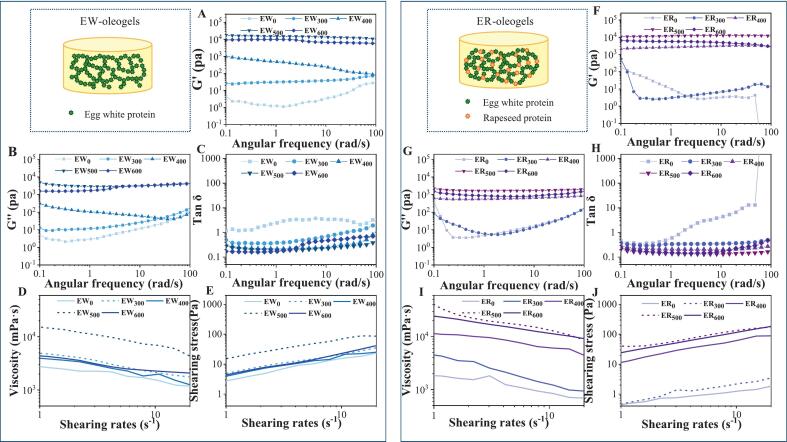
Rheological properties of EW oleogel (A-C) and ER oleogel (F-H). Viscosity curves and shear stress of EW oleogel (D-E) and ER oleogel (I-J). *Note:* EW is egg white protein. ER is egg white-rapeseed dual protein.

Oleogels exhibited varying degrees of mechanical stability upon inversion. To explain this phenomenon, CLSM was employed to visualize the microstructure. The oil phase of the oleogels was stained red by Nile Red, while proteins were stained green by Nile Blue [31]. As shown in Fig. 7B, without ultrasound treatment, the oleogels exhibited non-uniformly distributed voids of the oil and protein, which manifested as pronounced flow upon inversion. As ultrasound power increased, the oil phase became more homogeneous, along with a reduction in droplet size polydispersity. At 500 W, the ER oleogels exhibited the most uniform distribution of both oil and protein, and the gel network effectively immobilized oil droplets, resulting in the weakest macroscopic flow upon inversion. This observation aligns with the homogeneous oil distribution reported by Yu et al. [23] in soy protein isolate-phosphatidylserine walnut oil oleogels prepared at 450 W, suggesting that moderate ultrasound power promoted protein unfolding and subsequent network formation. However, at excessive ultrasound power (600 W), the ER oleogel exhibited a markedly porous microstructure, deteriorating the rheological properties and structural integrity (Fig. 8).

#### 3.4.2 Rheological properties of oleogels

Rheological properties are critical quality indicators for oleogels, providing insights into their gel network strength and viscoelastic behavior [56]. As shown in Fig. 8, within the frequency range of 0.1–100 rad/s, the storage modulus (G') and the loss modulus (G'') of EW_0_ and ER_0_ were unstable, with loss tangent (tan δ = G“/G') > 1. The G' of all ultrasound-treated samples exceeded G'', indicating that ultrasound treatment promoted the formation of a gel network with predominantly elastic, viscoelastic solid-like characteristics. This phenomenon likely stemmed from the increased lipophilicity of microgels induced by ultrasound-induced exposure of hydrophobic groups within protein molecules, which reinforced the three-dimensional network structure of the oleogel as evidenced by CLSM (Fig. 7). As shown in Fig. 8 A and F, the G' of ER oleogels and EW oleogels increased with increasing ultrasound power, peaking at 500 W. This finding aligns with the observations of Li et al. [24], who demonstrated that ultrasound treatment could significantly enhance the G' of pine oil oleogel and walnut oleogel prepared with wax as the oleogelators. Additionally, as shown in Fig. 8C and H, the tan δ of ER oleogel decreased with increasing ultrasound power, and ER_500_ reached a minimum. The increase in G' and decrease in tan δ of ER_500_ indicated that the ultrasound-induced structural modification of the dual-protein system contributed to improved oleogel quality. However, excessive ultrasound power induced protein over-aggregation, resulting in reduced surface hydrophobicity of the ER_600_ system (Fig. 2, Fig. 3), which in turn diminished the OAC of the ER_600_ microgel (Fig. 6C), consequently decreased the G' of ER_600_, and destabilized the oleogel structure [57].

As the shear rate increased, the viscosity of both ER oleogels and EW oleogels decreased (Fig. 8D and I), presenting a typical shear-thinning phenomenon. The high-speed shear disrupted the three-dimensional network structure of the oleogel [58]. Among the oleogels, ER_0_ exhibited lower viscosity and shear stress than EW_0_, suggesting that although the dual-protein oleogels had a higher storage modulus (G'), they showed poor structural stability under continuous shearing. This was likely attributable to weaker interfacial interactions between egg white and rapeseed proteins compared with those within egg white protein alone in the absence of ultrasound treatment. As a result, viscosity and shear stress were reduced. Similarly, Homolya et al. [59] reported that the BW15M5 sample in a beeswax/monoglyceride oleogel system displayed the highest G' yet the smallest deformation at the flow point, demonstrating that a highly elastic network structure underwent brittle fracture at relatively small deformations. Under shear, the oil droplets were rearranged along the flow direction, reducing flow resistance and resulting in a concomitant reduction in viscosity [60]. Additionally, Fig. 8D and I showed that ultrasound treatment enhanced the viscosity of oleogels, a phenomenon also observed by Liu et al. [61]. The increased viscosity likely stemmed from the enhanced OAC of the microgels induced by ultrasound treatment (Fig. 6C), facilitating a more compact oleogel structure (Fig. 7). As shown in Fig. 8E and J, the ultrasound-treated samples exhibited higher shear stress than untreated counterparts at equivalent shear rates, with values increasing as ultrasound power rose and peaking at 500 W. This indicated that ultrasound treatment enhanced the mechanical strength of the oleogel. However, excessive ultrasound power led to a decrease in shear stress in 600 W treated samples (EW_600_ and ER_600_ oleogels), consistent with the decline in G' described above, which could be attributed to protein over-aggregation.

## 4 Conclusion

This study found that the egg white-rapeseed (ER) dual-protein system incorporating rapeseed protein exhibited significantly higher surface hydrophobicity of the protein and the hydrogels, compared with the egg white (EW) system. The addition of rapeseed protein decreased the contact angle and increased the OAC. The increase in lipophilicity was attributed to the denser microstructure of the oleogel, leading to the G' of the oleogel increasing. Based on this, ultrasonic cavitation induced dual-protein unfolding and exposed hydrophobic groups. As ultrasound power increased from 0 W to 500 W, the surface hydrophobicity of ER_500_ reached the maximum, with the contact angle of ER_500_ microgel decreasing to a minimum of 31.97°. The exposure of hydrophobic groups improved the lipophilicity of the ER dual-protein microgel, which was proved by the OAC of ER_500_ microgel (196.00%) significantly higher than that of ER_0_ microgel (177.30%). In addition, the intense shear forces generated by cavitation disrupted protein aggregates, reducing microgel particle size, with ER_500_ achieving the smallest size (25.0 μm). The increased lipophilicity and reduced particle size of the microgels facilitated the formation of a dense network structure in the ER_500_ oleogels, leading to the G' and G" improvement. However, excessive ultrasound power (600 W) induced protein aggregation, increasing the particle size of ER_600_ microgel to 50.4 μm, which decreased the OAC of the microgel and reduced the G' of the oleogel. Overall, the addition of rapeseed protein coupled with moderate ultrasound treatment (500 W) improves the rheological properties and network structure of oleogel by enhancing protein hydrophobicity, reducing microgel particle size, and increasing lipophilicity. Future research will focus on validating the feasibility of substituting conventional solid fats with these oleogels in bakery applications (e.g., cookies and cakes) and systematically evaluating their impact on product texture, sensory attributes, and shelf-life stability.

## CRediT authorship contribution statement

**Zhenlian Liao:** Writing – original draft, Formal analysis. **Haojun Han:** Formal analysis. **Junkun Ma:** Methodology, Data curation. **Yisha Xie:** Writing – review & editing, Supervision, Funding acquisition. **Xi Cao:** Supervision, Funding acquisition.

## Declaration of competing interest

The authors declare that they have no known competing financial interests or personal relationships that could have appeared to influence the work reported in this paper.

## Appendix A Supplementary data

Supplementary Data 1 Supplementary Tables S1–S4: Pearson correlation analysis of the main evaluated indexes for ER and EW samples, and surface hydrophobicity parameters of EW/ER proteins and hydrogels.
