# Supplementary material for: Protein-based oleogels: effect of ultrasound on the structural characteristics of egg white-rapeseed dual-protein system
Source: Ultrason Sonochem. 2026 Aug 6;132:107992. doi: 10.1016/j.ultsonch.2026.107992 (PMC13487394; doi:10.1016/j.ultsonch.2026.107992)
Supplement: Supplementary file 1 [file mmc1.docx]

**Supplementary materials**

**Title: Protein-based oleogels: effect of ultrasound on the structural characteristics of rapeseed-egg white dual-protein system**

**Table S1** Pearson correlation analysis of the main evaluated indexes detected in the ER-sample.

|  | G'  (1 rad/s) | OAC | Contact angle | Particle size | Hydrophobicity of hydrogel | Hydrophobicity of protein |
| --- | --- | --- | --- | --- | --- | --- |
| G' (1 rad/s) | 1 | 0.519 | -0.445 | -0.959** | 0.352 | 0.716 |
| OAC |  | 1 | -0.69 | -0.697 | 0.202 | 0.373 |
| Contact angle |  |  | 1 | 0.51 | -0.804 | -0.82 |
| Particle size |  |  |  | 1 | -0.335 | -0.649 |
| hydrophobicity of hydrogel |  |  |  |  | 1 | 0.877 |
| hydrophobicity of protein |  |  |  |  |  | 1 |

*Note*. * Correlation is significant at the 0.05 level (2-tailed). ** Correlation is significant at the 0.01 level (2-tailed).

**Table S2** Pearson correlation analysis of the main evaluated indexes detected in the EW-sample.

|  | G'  (1 rad/s) | OAC | Contact angle | Particle size | Hydrophobicity of hydrogel | Hydrophobicity of protein |
| --- | --- | --- | --- | --- | --- | --- |
| G' (1 rad/s) | 1 | 0.563 | -0.399 | -0.103 | 0.815 | 0.894* |
| OAC |  | 1 | -0.798 | -0.164 | 0.688 | 0.776 |
| Contact angle |  |  | 1 | -0.454 | -0.795 | -0.439 |
| Particle size |  |  |  | 1 | 0.295 | -0.344 |
| hydrophobicity of hydrogel |  |  |  |  | 1 | 0.669 |
| hydrophobicity of protein |  |  |  |  |  | 1 |

*Note*. * Correlation is significant at the 0.05 level (2-tailed). ** Correlation is significant at the 0.01 level (2-tailed).

**Table S3** The λ_max_ and F_max_ parameters of surface hydrophobicity of EW and ER proteins under different ultrasound powers.

| Protein | λ_max_ (nm) | F_max_ (a.u.) |
| --- | --- | --- |
| EW_0_ | 490±6 ^a^ | 87924.49±536.24 ^c^ |
| EW_300_ | 487±4 ^a^ | 100665.29±2947.85 ^b^ |
| EW_400_ | 486±2 ^a^ | 99277.46±1860.82 ^b^ |
| EW_500_ | 483±4 ^a^ | 119160.40±3500.49 ^a^ |
| EW_600_ | 481±1 ^a^ | 125449.27±240.60 ^a^ |
| ER_0_ | 479±2 ^a^ | 124623.55±3995.17 ^c^ |
| ER_300_ | 477±1 ^a^ | 125312.28±660.77 ^c^ |
| ER_400_ | 477±1 ^a^ | 136610.50±2970.06 ^b^ |
| ER_500_ | 475±2 ^a^ | 157409.99±3298.12 ^a^ |
| ER_600_ | 475±1 ^a^ | 143377.38±4423.18 ^b^ |

*Note*. Different lowercase letters indicate significant differences among ultrasound powers within the same group (P < 0.05)

**Table S4** The λ_max_ and F_max_ parameters of surface hydrophobicity of EW and ER hydrogel under different ultrasound powers.

| Hydrogel | λ_max_ (nm) | F_max_ (a.u.) |
| --- | --- | --- |
| EW_0_ | 480±3 ^a^ | 54642.06±1379.82 ^b^ |
| EW_300_ | 481±2 ^a^ | 54576.99±885.03 ^b^ |
| EW_400_ | 479±2 ^a^ | 58460.76±378.11 ^b^ |
| EW_500_ | 479±1 ^a^ | 65589.16±3870.37 ^a^ |
| EW_600_ | 477±6 ^a^ | 56056.68±3421.10 ^b^ |
| ER_0_ | 480±2 ^a^ | 61686.46±1100.05 ^c^ |
| ER_300_ | 477±3 ^a^ | 74956.56±1089.49 ^b^ |
| ER_400_ | 475±3 ^a^ | 77281.28±1563.56 ^b^ |
| ER_500_ | 475±3 ^a^ | 88100.20±3273.19 ^a^ |
| ER_600_ | 476±2 ^a^ | 81213.79±3284.81 ^ab^ |

*Note*. Different lowercase letters indicate significant differences among ultrasound powers within the same group (P < 0.05)
